# Population Pharmacokinetic Properties of Antituberculosis Drugs in Vietnamese Children with Tuberculous Meningitis

**DOI:** 10.1128/AAC.00487-20

**Published:** 2020-12-16

**Authors:** Navarat Panjasawatwong, Thanaporn Wattanakul, Richard M. Hoglund, Nguyen Duc Bang, Thomas Pouplin, Wichit Nosoongnoen, Vi Nguyen Ngo, Jeremy N. Day, Joel Tarning

**Affiliations:** a Faculty of Pharmacy, Mahidol University, Bangkok, Thailand; b Mahidol-Oxford Tropical Medicine Research Unit, Faculty of Tropical Medicine, Mahidol University, Bangkok, Thailand; c Centre for Tropical Medicine and Global Health, Nuffield Department of Medicine, University of Oxford, Oxford, United Kingdom; d Oxford University Clinical Research Unit, Ho Chi Minh City, Vietnam; e Pham Ngoc Thach Hospital, Ho Chi Minh City, Vietnam

**Keywords:** population pharmacokinetics, antituberculosis drugs, dose optimization, pediatric, tuberculosis meningitis

## Abstract

Optimal dosing of children with tuberculous meningitis (TBM) remains uncertain and is currently based on the treatment of pulmonary tuberculosis in adults. This study aimed to investigate the population pharmacokinetics of isoniazid, rifampin, pyrazinamide, and ethambutol in Vietnamese children with TBM, to propose optimal dosing in these patients, and to determine the relationship between drug exposure and treatment outcome. A total of 100 Vietnamese children with TBM were treated with an 8-month antituberculosis regimen. Nonlinear mixed-effects modeling was used to evaluate the pharmacokinetic properties of the four drugs and to simulate different dosing strategies. The pharmacokinetic properties of rifampin and pyrazinamide in plasma were described successfully by one-compartment disposition models, while those of isoniazid and ethambutol in plasma were described by two-compartment disposition models. All drug models included allometric scaling of body weight and enzyme maturation during the first years of life. Cerebrospinal fluid (CSF) penetration of rifampin was relatively poor and increased with increasing protein levels in CSF, a marker of CSF inflammation. Isoniazid and pyrazinamide showed good CSF penetration. Currently recommended doses of isoniazid and pyrazinamide, but not ethambutol and rifampin, were sufficient to achieve target exposures. The ethambutol dose cannot be increased because of ocular toxicity. Simulation results suggested that rifampin dosing at 50 mg/kg of body weight/day would be required to achieve the target exposure. Moreover, low rifampin plasma exposure was associated with an increased risk of neurological disability. Therefore, higher doses of rifampin could be considered, but further studies are needed to establish the safety and efficacy of increased dosing.

## Abstract (precis)

Optimal dosing of children with tuberculous meningitis (TBM) remains uncertain and is currently based on the treatment of pulmonary tuberculosis in adults. This study aimed to investigate the population pharmacokinetics of isoniazid, rifampin, pyrazinamide, and ethambutol in Vietnamese children with TBM, to propose optimal dosing in these patients, and to determine the relationship between drug exposure and treatment outcome. A total of 100 Vietnamese children with TBM were treated with an 8-month antituberculosis regimen.

## TEXT

Tuberculosis (TB), caused by the bacillus Mycobacterium tuberculosis, is a major health problem in many countries. In 2018, approximately 10 million people worldwide were diagnosed with new TB infections and most of these were in Southeast Asia (43.7%) and Africa (24.5%). Of the 10 million newly infected TB patients, 15% had extrapulmonary TB (1). Tuberculous meningitis (TBM) is the most severe form of extrapulmonary TB, triggered by invasion by the pathogen of the membranes and fluid surrounding the brain and spinal cord and of the brain/cord parenchyma itself (2). Even on current standard treatment, the mortality rate of TBM is 30%, with half of survivors suffering long-term neurological sequelae (3). Like other forms of disseminated tuberculosis, TBM is more common in children below 5 years of age and in immunosuppressed persons (4).

The first-line anti-TB regimen consists of four drugs (isoniazid, rifampin, pyrazinamide, and ethambutol) which play different roles in the course of treatment. Significant between-patient variability has been described in isoniazid plasma concentrations, driven by polymorphisms in the NAT2 gene. This gene encodes the enzyme N-acetyltransferase 2 (NAT2), which metabolizes isoniazid, and the polymorphisms determine the rate with which this occurs (5). Meanwhile, the metabolism of rifampin is complicated by the fact that it has autoinduction properties (6). There is a time-dependent upregulation of the enzyme responsible for its metabolism which impacts drug levels. In addition, rifampin exhibits concentration-dependent clearance, i.e., a saturation of elimination processes at higher rifampin concentrations (7). Optimal dosing of children with TBM is uncertain and largely based on data derived from the treatment of pulmonary TB in adults. Several studies have shown that plasma concentrations of the four first-line anti-TB drugs were lower in children than in adults after administration of standard weight-based doses (mg/kg of body weight) of anti-TB drugs (8–13). Thus, extrapolation of adult dosages to children might result in an increased risk of suboptimal treatment outcome and development of drug resistance.

The lower exposure observed in children can be explained by several factors, such as body weight, body composition, maturation level of metabolizing enzymes and transporters, and organ function. The pediatric dose recommendations from the World Health Organization (WHO) in 2006 of isoniazid 5 mg/kg, rifampin 10 mg/kg, pyrazinamide 25 mg/kg, and ethambutol 15 mg/kg daily (14) were demonstrated to be suboptimal in several studies (15, 16). For example, the median steady-state peak concentration of rifampin was 10 mg/liter in adults (15) and 5.1 mg/liter in children (16) after administration of rifampin at 10 mg/kg/day. Those studies showed that 42% of adults and 77% of children had rifampin peak concentrations below the therapeutic level of 8 mg/liter (17). Due to those findings, current pediatric dose recommendations from the WHO in 2014 have been increased, i.e., isoniazid 10 mg/kg, rifampin 15 mg/kg, pyrazinamide 35 mg/kg, and ethambutol 20 mg/kg daily (14). Still, this increased dosing has been shown to fail to achieve therapeutic drug concentrations in certain pediatric studies (18–20).

Population pharmacodynamic (PD) analyses in adults with TBM showed that rifampin plasma exposure (area under the concentration-time curve from 0 to 24 h [AUC_0–24_]) was a predictor of survival (21, 22). The values for rifampin plasma AUC_0–24_, corresponding to 50% of maximal survival (50% effective concentration [EC_50_]), reported in those two studies were 86.4 mg×h/liter (21) and 171 mg×h/liter (22). Another study demonstrated that isoniazid exposure in plasma and cerebrospinal fluid (CSF) was associated with the risk of death in adults with TBM, estimating the EC_50_ to be 7.03 mg×h/liter in both plasma and CSF (23). To date, no association between outcome and pyrazinamide and ethambutol exposures has been established in patients with TBM.

Although the population pharmacokinetic (PK) properties of anti-TB drugs have been reported for children with pulmonary TB, there is still limited information available for children with TBM. Furthermore, very limited information is available regarding children with respect to the penetration of these drugs into CSF, an important consideration given that the site of action of TBM is in the brain and meninges. In addition, data from this study consist of both PK and PD in TBM children, which is rare. The objectives of this study were to (i) investigate the population PK properties of the four first-line anti-TB drugs in plasma and CSF in Vietnamese children with TBM, (ii) propose an optimal dosage regimen of the anti-TB drugs in these children, and (iii) determine the relationship between drug exposures and treatment outcomes.

## RESULTS

### Demographic data.

One hundred Vietnamese children with suspected TBM were enrolled in the study. Fifty-six subjects (56%) were boys, and the median age was 3 years. Demographic data and baseline laboratory tests are summarized in Table 1.

**TABLE 1:** Demographic and baseline data in children with TBM (*n* = 100)

Parameter	Values^*a*^
Clinical covariate	
Gender	
Boys	56 (56)
Girls	44 (44)
Body wt (kg)	10.9 (4.0 to 43)
Age (yrs)	3.0 (0.167 to 15.0)
Wt-for-age z-score	−1.93 (−5.52 to 1.99)
Ht-for-age z-score	−1.64 (−9.17 to 2.21)
Disease severity^*b*^	
Grade I	58 (58)
Grade II	24 (24)
Grade III	18 (18)
HIV infection	
HIV positive	4 (4)
HIV negative	92 (92)
Unknown	4 (4)
NAT2 acetylator status	
Fast	17 (17)
Intermediate	47 (47)
Slow	28 (28)
Unknown	8 (8)
C-reactive protein (mg/liter)	9.17 (0.04 to 113); [<3]

CSF laboratory tests	
White cell count (no. of cells/μl)	159 (1 to 2,950); [<5]
Neutrophil count (no. of cells/μl)	30.0 (0 to 90.0); [0]
Lymphocyte count (no. of cells/μl)	80.0 (10.0 to 100); [60 to 70]
Protein (g/liter)	1.20 (0.1 to 5); [<0.4]
Lactate (mmol/liter)	5.30 (1.21 to 19.2); [1 to 2]
CSF/plasma glucose ratio	0.262 (0.065 to 9.59); [≥0.6]

Therapeutic outcome	
Survival^*c*^	
Complete recovery	54 (54)
Intermediate disability	21 (21)
Severe disability	6 (6)
Death	15 (15)
Lost to follow-up	4 (4)

a Data are presented as number (percent) for categorical data and as median (min-max) for continuous data; data in square brackets represent normal laboratory range based on the levels used clinically at the Pham Ngoc Thach Hospital for Tuberculosis and Lung Diseases (PNT) in Ho Chi Minh City, Vietnam, in which the clinical trial was conducted.

b Disease severity was based on BCS for children <5 years and GCS for children ≥5 years. Grade I, BCS of 4 to 5 with no focal neurological signs or GCS of 15 with no focal neurological signs; grade II, BCS of 2 to 3 or BCS of 4 to 5 with focal neurological signs or GCS of 11 to 14 or GCS of 15 with focal neurological signs; grade III, BCS of ≤1 or GCS of ≤10.

c Therapeutic survival outcome data were stratified on the basis of the modified Rankin scale.

### Pharmacokinetic properties of isoniazid.

A total of 523 plasma and 140 CSF isoniazid concentrations were included in the pharmacokinetic analysis. A total of 48 plasma concentrations (9.2%) were below the limit of quantification (LOQ = 12 μg/liter). The M3 method was used to handle the data below the LOQ to avoid structural model misspecification. Pharmacokinetic properties of isoniazid were best described by a two-compartment disposition model, resulting in a significantly improved model fit compared to a one-compartment disposition model (delta objective function value [ΔOFV] = −48.2). Addition of another disposition compartment did not result in a significant improvement (ΔOFV = −1.71). Isoniazid absorption was described by two fixed-transit absorption compartments (the absorption rate constant [*k_a_*] value was set to be identical to the transfer rate constant [*k*_tr_] value), resulting in substantial improvement in model fit compared to other absorption models. No statistically significant improvement was seen when interoccasional variability (IOV) was implemented with respect to relative bioavailability or mean transit time (ΔOFV = −0.466). A CSF compartment was added to the final structural model, and the distribution (partition coefficient [PC]) from the central compartment to the CSF compartment was estimated to be 165% (95% confidence interval [CI], 148% to 181%). This indicates that isoniazid is actively transported over the blood-brain barrier, resulting in higher drug concentrations in CSF than in plasma. The fraction of unbound drug in plasma was fixed to 90% according to the value previously reported in the literature (24).

Body weight was incorporated as an allometric function on all clearance and volume-of-distribution parameters, resulting in improved model fit (ΔOFV = −43.0). Age-based enzyme maturation was found to be a significant covariate on clearance (ΔOFV = −17.6). The postmenstrual age at which 50% maturation of clearance occurred (MAT_50_) was 12.7 months (i.e., 3.37 months postpartum), resulting in 90% maturation at approximately 12 months postpartum (see Fig. S1 in the supplemental material). On the basis of genotype, 17 patients were predicted to be fast acetylators, 47 to be intermediate acetylators, and 28 to be slow acetylators. Eight patients did not complete the NAT2 genotyping and were therefore designated intermediate acetylators (i.e., the most common phenotype subgroup). The effect of NAT2 phenotype on isoniazid clearance was initially investigated using all three subgroups (i.e., fast, intermediate, and slow acetylators). The model estimated that clearance in the fast acetylator group was negligibly higher than in the intermediate group (8.57%). Thus, the use of a reduced covariate model, employing only two subgroups of acetylator statuses (i.e., fast acetylators [comprising fast, intermediate, and unknown acetylators, *n* = 72] and slow acetylators [*n* = 28]), resulted in model fit results similar to that obtained using three subgroups (ΔOFV = 2.03). The reduced covariate model demonstrated that NAT2 phenotype was a significant covariate on clearance of isoniazid (ΔOFV = −37.0), resulting in 56.4% (95% CI, 49.0% to 63.4%) reduced clearance in patients with slow acetylator status compared to those with fast acetylator status. No other covariates had a significant impact. Interindividual variability (IIV) was supported on clearance (36.8%) and intercompartmental clearance (101%). Additive residual error models on the logarithmic scale were used for both plasma and CSF isoniazid concentrations.

**FIG 1:**
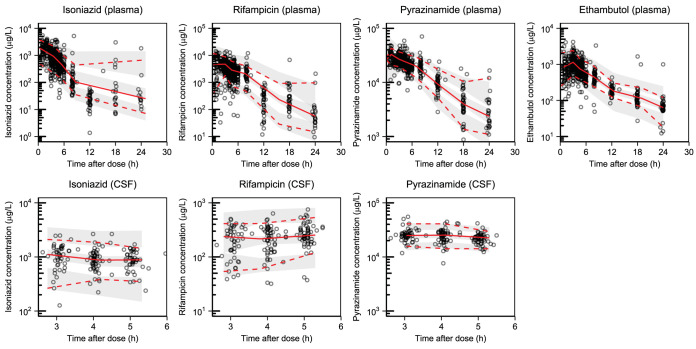
Prediction-corrected visual predictive checks of the final population pharmacokinetic models describing plasma (top panels) and CSF (bottom panels) isoniazid, rifampin, pyrazinamide, and ethambutol. Open circles represent the observed data. The lower, middle, and upper lines represent the 5th, 50th, and 95th percentiles of the observed data. The shaded areas represent the 95% confidence intervals of the 5th, 50th, and 95th percentiles of the simulated data (*n* = 1,000).

Population PK parameter estimates and *post hoc* secondary PK parameters from the final model are shown in Table 2. Simulation-based diagnostics (prediction-corrected visual predictive checks [pcVPCs]) indicated good overall predictive performance of the final model (Fig. 1), as did stratification by acetylator status (Fig. S2). The η-shrinkages of clearance and intercompartmental clearance were estimated to be 12.7% and 38.3%, respectively. The ε-shrinkages of plasma and CSF were 11.3% and 13.2%, respectively.

**TABLE 2:** Population PK parameter estimates and *post hoc* secondary PK parameters from the final population PK model of isoniazid in children with TBM^*a*^

Parameter	Population estimate^*b*^ (% RSE)^*c*^	95% CI^*c*^	% CV for IIV^*b*^ (% RSE)^*c*^	95% CI for IIV^*c*^	Plasma^*d*^	CSF^*d*^
Primary PK parameters						
*F*	1 (fixed)					
CL/*F* (liters/h)	9.43 (6.55)	8.33–10.7	36.8 (10.2)	30.6–45.7		
Vc/*F* (liters)	3.78 (33.6)	1.32–6.29				
MTT (h)	0.878 (10.9)	0.682–1.05				
MAT_50_ (mo)	12.7 (6.85)	10.9–14.3				
HILL	4.7 (13.5)	3.60–6.04				
*Q*/*F* (liters/h)	28.0 (21.5)	18.2–41.3	101 (11.7)	69.6–133		
Vp/*F* (liters)	15.3 (9.60)	12.6–18.3				
*Q*_CSF_/*F* (liters/h)	13.7 (43.6)	4.45–27.5				
*f*_u_	0.9 (fixed)					
PC	1.65 (5.24)	1.48–1.81				
Slow acetylators (%)	56.4 (6.61)	49.0–63.4				
σ_Plasma_	0.474 (4.18)	0.402–0.556				
σ_CSF_	0.170 (6.96)	0.129–0.220				

Secondary PK parameters at steady state						
Fast and intermediate acetylators						
AUC_0–24_ (mg×h/liter)					6.35 (2.64–24.2)	9.42 (3.91–35.9)
*C*_max_ (mg/liter)					2.12 (1.47–5.13)	3.14 (2.18–7.61)
*T*_max_ (h)					0.942 (0.801–1.19)	0.955 (0.821–1.19)
*t*_1/2_ (h)					2.14 (1.04–6.24)	2.14 (1.04–6.24)
Slow acetylators						
AUC_0–24_ (mg×h/liter)					12.4 (6.38–21.6)	18.4 (9.47–32.1)
*C*_max_ (mg/liter)					2.41 (1.88–3.84)	3.58 (2.79–5.70)
*T*_max_ (h)					1.14 (0.890–4.52)	1.15 (0.920–4.53)
*t*_1/2_ (h)					3.66 (2.08–6.38)	3.66 (2.08–6.38)

a Abbreviations: *F*, relative bioavailability; CL/*F*, oral elimination clearance; Vc/*F*, volume of distribution of the central compartment; MTT, mean transit absorption time; MAT_50_, postmenstrual age at which clearance is 50% of the mature clearance; HILL, Hill coefficient for maturation clearance; *Q*/*F*, intercompartmental clearance; Vp/*F*, volume of distribution of peripheral compartment; *Q*_CSF_/*F*, intercompartmental clearance between central and CSF compartments; *f*_u_, fraction unbound; PC, transfer multiplier between central and CSF compartments describing blood-brain penetration; Slow acetylator, reduction of elimination clearance (percent) in slow acetylator compared to fast acetylator; IIV, interindividual variability; σ, variance of the residual variability, incorporated as an additive error on the logarithmic scale; AUC_0–24_, area under the concentration-time curve from 0 to 24 h; *C*_max_, peak concentration; *T*_max_, time to reach peak concentration; *t*_1/2_, terminal elimination half-life.

b Computed population mean parameter estimates from NONMEM. Parameter estimates are scaled to typical patient at 10.9 kg and 3 years.

c Assessed by sampling importance resampling (SIR).

d Median (min-max).

### Pharmacokinetic properties of rifampin.

A total of 512 plasma and 155 CSF rifampin concentrations were used in the model development. A total of 26 plasma samples (5.08%) were below the LOQ (LOQ = 8.0 μg/liter) and coded as missing data. Rifampin pharmacokinetic properties were best described by a one-compartment disposition model. No further improvement was seen with a two-compartment disposition model (ΔOFV = −1.66). Absorption of rifampin was characterized by two fixed-transit absorption compartments (*k_a_* was set to be identical to *k*_tr_), resulting in a substantially improved model fit compared to other absorption models tested. Incorporation of IOV on relative bioavailability or mean transit time did not improve the model fit significantly (ΔOFV = −1.02). A CSF compartment was added to the final structural model, resulting in 84.4% (95% CI, 72.7% to 98.0%) of unbound rifampin distributing from the central compartment to the CSF compartment. The fraction of unbound drug in plasma was fixed to 20% on the basis of the value previously reported in the literature, resulting in only 16.9% (95% CI, 14.5% to 19.6%) of total drug penetrating the blood-brain barrier (25, 26).

**FIG 2:**
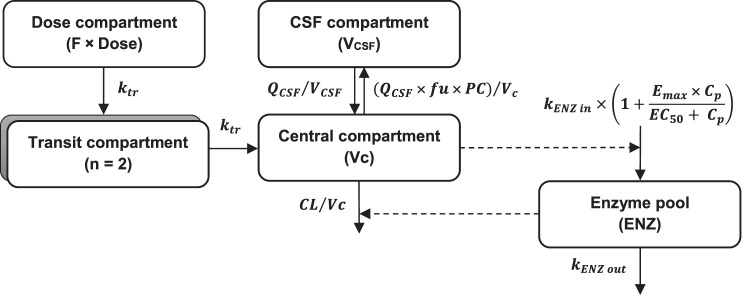
Final rifampin pharmacokinetic model. Abbreviations: *Vc*, central volume of distribution; *V_CSF_*, volume of distribution of CSF compartment; *k_tr_*, transfer rate constant; *Q_CSF_*, intercompartmental clearance between central and CSF compartments; *fu*, fraction unbound; *PC*, penetration fraction from central to CSF compartment; *K_ENZ in_*, zero-order enzyme production rate; *K_ENZ out_*, first-order rate constant of enzyme degradation; *E_max_*, maximum enzyme induction; *EC*_50_, drug concentration that results in half of *E_max_*; ENZ, the amount of metabolizing enzyme in the enzyme pool; F, relative bioavailability; *C_p_*, plasma concentration.

Body weight was implemented as an allometric function on all clearance and volume of distribution parameters, resulting in improved model fit (ΔOFV = −64.4). Enzyme maturation was found to be a significant covariate on clearance (ΔOFV = −7.56). The MAT_50_ was 6.81 months, corresponding to 90% maturation at 36 months postpartum (Fig. S1). The concentration of protein in CSF was found to be a statistically significant covariate on PC (ΔOFV = −36.0). The relationship between CSF protein concentration and the PC was best described by an exponential function; i.e., an increase of 1 g/liter in CSF protein concentration resulted in a 1.28-fold increase in PC. The link between CSF protein concentration and CSF AUC_0–24_ is illustrated in Fig. S3. Implementing an enzyme turnover model (i.e., enzyme autoinduction) with parameters fixed to those reported in a previously published study in adults (6) resulted in a substantially improved model fit (ΔOFV = −91.9). Estimation of these enzyme turnover parameters yielded unreasonable results (EC_50_ was estimated to be 1.01 μg/liter, whereas the measured rifampin concentrations in this study were between 9.13 and 16,400 μg/liter). There were not enough data collected in this study to estimate the previously proposed concentration-dependent nonlinear elimination. Furthermore, the implementation of nonlinear elimination, using fixed nonlinear elimination parameters (i.e., *K_m_* and *V*_max_) corresponding to previously published values (7), did not improve the model fit. No other covariates had a significant impact. The final model included IIV on clearance (19.4%), volume of distribution of the central compartment (23.0%), mean transit time (85.0%), and PC (22%). Additive residual error models on the logarithmic scale were used for both plasma and CSF rifampin concentrations. The final rifampin pharmacokinetic model is illustrated in Fig. 2.

Population PK parameter estimates and *post hoc* secondary PK parameters from the rifampin final model are presented in Table 3. Simulation-based diagnostics (pcVPCs) indicated good predictive performance of the final model (Fig. 1). The η-shrinkages of the clearance, central volume of distribution, mean transit absorption time, and PC were estimated to be 40.4%, 48.2%, 28.1%, and 58.7%, respectively. The ε-shrinkages of plasma and CSF were calculated to be 10.4% and 9.77%, respectively.

**TABLE 3:** Population PK parameter estimates and *post hoc* secondary PK parameters from the final population PK model of rifampin in children with TBM^*a*^

Parameter	Population estimate^*b*^ (% RSE)	95% CI^*c*^	% CV for IIV^*b*^ (% RSE^*c*^)	95% CI for IIV^*c*^	Plasma^*d*^	CSF^*d*^
Primary PK parameters						
*F*	1 (fixed)					
CL/*F* (liters/h)	3.22 (8.94)	2.83–3.95	19.4 (16.3)	14.5–26.4		
Vc/*F* (liters)	12.3 (6.46)	10.9–13.9	23.0 (11.6)	16.3–27.6		
MTT (h)	1.25 (13.0)	0.921–1.55	85.0 (11.9)	64.7–117		
MAT_50_ (mo)	6.81 (25.0)	3.27–9.87				
HILL	1.38 (33.0)	0.584–2.28				
*K*_enz_ (h^−1^)	0.00369 (fixed)					
*E*_max_	1.04 (fixed)					
EC_50_ (μg/liter)	70.5 (fixed)					
*Q*_CSF_/*F* (liters/h)	0.00482 (11.6)	0.00368–0.00582				
*f*_u_	0.2 (fixed)					
PC	0.844 (7.84)	0.727–0.980	22.0 (18.6)	12.4–29.2		
CSF protein on PC (%)	24.5 (17.6)	16.5–32.9				
σ_Plasma_	0.513 (4.10)	0.439–0.608				
σ_CSF_	0.309 (8.12)	0.229–0.427				

Secondary PK parameters at steady state						
AUC_0–24_ (mg×h/liter)					21.5 (14.2–36.5)	4.08 (2.59–9.34)
*C*_max_ (mg/liter)					4.94 (2.46–8.37)	0.253 (0.161–0.559)
*T*_max_ (h)					2.13 (0.406–5.71)	10.0 (5.72–13.9)
*t*_1/2_ (h)					3.09 (1.45–6.53)	3.09 (1.45–6.53)

a Abbreviations: *F*, relative bioavailability; CL/*F*, oral elimination clearance; Vc/*F*, volume of distribution of the central compartment; MTT, mean transit absorption time; MAT_50_, postmenstrual age at which clearance is 50% of the mature clearance; HILL, Hill coefficient for maturation clearance; *K*_enz_, first-order rate constant of enzyme degradation; *E*_max_, maximum enzyme induction; EC_50_, drug concentration that results in half of *E*_max_; *Q*_CSF_/*F*, intercompartmental clearance between central and CSF compartments; *f*_u_, fraction unbound; PC, transfer multiplier between central and CSF compartments describing blood-brain penetration; IIV, interindividual variability; σ, variance of the residual variability, incorporated as an additive error on the logarithmic scale; AUC_0–24_, area under the concentration-time curve from 0 to 24 h; *C*_max_, peak concentration; *T*_max_, time to reach peak concentration; *t*_1/2_, terminal elimination half-life.

b Computed population mean parameter estimates from NONMEM. Parameter estimates are scaled to typical patient at 10.9 kg and 3 years.

c Assessed by sampling importance resampling (SIR).

d Median (min-max).

### Pharmacokinetic properties of pyrazinamide.

For pyrazinamide, 519 plasma and 155 CSF concentrations were used for model development. A total of 11 plasma samples (2.12%) were below the LOQ (LOQ = 800 μg/liter) and were coded as missing data. The pharmacokinetic properties were well described by a one-compartment disposition model. A two-compartment disposition model did not improve the model fit (ΔOFV = −0.174). The absorption phase was described by three fixed-transit absorption compartments (*k_a_* was set to be identical to *k*_tr_), which resulted in a better fit than was seen with the other absorption models investigated. IOV of the relative bioavailability was statistically significant (ΔOFV = −9.45) and estimated to be 19.0%. A CSF compartment was added to the final structural model, and the distribution of unbound drug was estimated to be close to 100%, indicating good CSF penetration. The fraction of unbound drug in plasma was fixed to 90% based on the value previously reported in the literature (25, 26).

Inclusion of body weight as an allometric function, and of age as a maturation function, improved the model fit substantially (ΔOFV of −247 and −37.0, respectively). The MAT_50_ was 12.1 months, resulting in enzyme systems in children reaching 90% of those in adults at 18 months postpartum (Fig. S1). Weight-for-age z-score (WAZ) was a significant covariate on the clearance and the central volume of distribution (ΔOFV = −19.4). No other covariates had a significant impact. IIV was supported on clearance (20.0%), volume of distribution of the central compartment (18.3%), and mean transit time (64.5%). Additive residual error models on the logarithmic scale were used for both plasma and CSF pyrazinamide concentrations.

Population PK parameter estimates and *post hoc* secondary PK parameters from the final pyrazinamide model are presented in Table 4. Simulation-based diagnostics (pcVPCs) indicated good predictive performance of the final model (Fig. 1). The η-shrinkages for clearance, central volume of distribution, and mean transit time were estimated to be 17.8%, 28.3%, and 48.8%, respectively. The ε-shrinkages of plasma and CSF were calculated to be 28.4% and 38.0%, respectively.

**TABLE 4:** Population PK parameter estimates and *post hoc* secondary PK parameters from the final population PK model of pyrazinamide in children with TBM^*a*^

Parameter	Population estimate^*b*^ (% RSE)	95% CI^*c*^	% CV for IIV^*b*^ (% RSE^*c*^)	95% CI for IIV^*c*^	% CV for IOV^*b*^ (% RSE^*c*^)	95% CI for IOV^*c*^	Plasma^*d*^	CSF^*d*^
Primary PK parameters								
*F*	1 (fixed)				19.0 (7.05)	16.5–21.8		
CL/*F* (liters/h)	1.07 (4.02)	0.996–1.17	20.0 (6.95)	17.0–22.6				
Vc/*F* (liters)	7.38 (2.73)	6.99–7.80	18.3 (10.6)	14.2–22.1				
MTT (h)	0.457 (10.8)	0.364–0.551	64.5 (12.0)	48.7–83.8				
MAT_50_ (mo)	12.1 (7.26)	10.2–13.7						
HILL	2.73 (29.0)	1.63–4.64						
*Q*_CSF_/*F* (liters/h)	0.0964 (13.6)	0.0779–0.129						
*f*_u_	0.9 (fixed)							
PC	1.02 (1.70)	0.989–1.06						
WAZ on CL/*F* (%)	4.76 (18.3)	3.36–6.68						
WAZ on Vc/*F* (%)	−4.65 (27.6)	−6.95– −1.98						
σ_Plasma_	0.0274 (4.97)	0.0230–0.0335						
σ_CSF_	0.0114 (12.8)	0.00652–0.0181						
								
Secondary PK parameters at steady state								
AUC_0–24_ (mg×h/liter)							288 (108–569)	266 (98.9–522)
*C*_max_ (mg/liter)							42.5 (29.8–92.7)	31.3 (19.4–72.2)
*T*_max_ (h)							1.08 (0.259–2.27)	3.06 (2.17–5.29)
*t*_1/2_ (h)							5.12 (3.01–14.5)	5.12 (3.01–14.5)

a Abbreviations: *F*, relative bioavailability; CL/*F*, oral elimination clearance; Vc/*F*, volume of distribution of the central compartment; MTT, mean transit absorption time; MAT_50_, postmenstrual age at which clearance is 50% of the mature clearance; HILL, Hill coefficient for maturation clearance; *Q*_CSF_/*F*, intercompartmental clearance between central and CSF compartments; *f*_u_, fraction unbound; PC, transfer multiplier between central and CSF compartments describing blood-brain penetration; WAZ, weight-for-age z-score; IIV, interindividual variability; IOV, interoccasion variability; σ, variance of the residual variability, incorporated as an additive error on the logarithmic scale; AUC_0–24_, area under the concentration-time curve from 0 to 24 h; *C*_max_, peak concentration; *T*_max_, time to reach peak concentration; *t*_1/2_, terminal elimination half-life.

b Computed population mean parameter estimates from NONMEM. Parameter estimates are scaled to typical patient at 10.9 kg and 3 years.

c Assessed by sampling importance resampling (SIR).

d Median (range).

### Pharmacokinetic properties of ethambutol.

The pharmacokinetic model for ethambutol was developed using 517 plasma concentrations. No CSF concentrations were available due to bioanalytical issues (see Materials and Methods). A total of 1 plasma sample (0.193%) was below the LOQ (LOQ = 8 μg/liter) and coded as missing data. A two-compartment disposition model was superior to a one-compartment disposition model (ΔOFV = −74.6). The absorption phase was well described by two fixed-transit compartments (*k_a_* was set to be identical to *k_tr_*), resulting in a substantially improved model fit compared to other absorption models. Incorporation of IOV on relative bioavailability or mean transit time did not improve the model fit significantly (ΔOFV of −2.32 or −3.27, respectively).

The addition of body weight as an allometric function and age as a maturation function improved the model fit substantially (ΔOFV of −100 and −6.58, respectively). The estimated Hill parameter in the enzyme maturation function (i.e., Hill parameter value of 76.1) resulted in a fully mature enzyme system at 3 months postpartum, which is biologically implausible. Therefore, the Hill parameter was fixed to 1 in the final model, resulting in an estimated MAT_50_ of 3.99 months, corresponding to 90% of matured enzyme systems at approximately 30 months postpartum (Fig. S1). No other covariates had a significant impact. The final model included IIV on relative bioavailability (20.1%), volume of distribution of the central compartment (51.1%), mean transit time (18.4%), and volume of distribution of peripheral compartment (96.3%). An additive residual error model on the logarithmic scale was used for plasma ethambutol concentrations.

Population PK parameter estimates and *post hoc* secondary PK parameters from the final ethambutol model are presented in Table 5. Simulation-based diagnostics (pcVPCs) indicated a good predictive performance of the final model (Fig. 1). The η-shrinkages of the central volume of distribution, relative bioavailability, peripheral volume of distribution, and mean transit time values were estimated to be 33.0%, 33.2%, 47.5%, and 61.8%, respectively. The ε-shrinkage was calculated to be 13.2%.

**TABLE 5:** Population PK parameter estimates and *post hoc* secondary PK parameters from the final population PK model of ethambutol in children with TBM^*a*^

Parameter	Population estimate^*b*^ (% RSE)	95% CI^*c*^	% CV for IIV^*b*^ (% RSE^*c*^)	95% CI for IIV^*c*^	Plasma^*d*^
Primary PK parameters					
*F*	1 (fixed)		20.1 (14.2)	14.3–25.7	
CL/*F* (liters/h)	28.2 (4.68)	25.7–30.8			
Vc/*F* (liters)	98.6 (11.0)	79.1–122	51.1 (10.7)	39.7–63.6	
MTT (h)	1.8 (7.15)	1.56–2.05	18.4 (21.3)	8.60–25.5	
MAT_50_ (mo)	3.99 (36.0)	1.60–7.20			
HILL	1 (fixed)				
*Q*/*F* (liters/h)	16.9 (12.2)	12.9–20.9			
Vp/*F* (liters)	153 (16.0)	108–203	96.3 (14.2)	63.2–134	
σ_Plasma_	0.197 (4.45)	0.166–0.236			

Secondary PK parameters at steady state					
AUC_0–24_ (mg×h/liter)					8.18 (4.60–17.8)
*C*_max_ (mg/liter)					1.26 (0.656–2.54)
*T*_max_ (h)					2.59 (1.91–3.65)
*t*_1/2_ (h)					12.7 (3.92–21.4)

a Abbreviations: *F*, relative bioavailability; CL/*F*, oral elimination clearance; Vc/*F*, volume of distribution of the central compartment; MTT, mean transit absorption time; MAT_50_, postmenstrual age at which clearance is 50% of the mature clearance; HILL, Hill coefficient for maturation clearance; *Q*/*F*, intercompartmental clearance; Vp/*F*, volume of distribution of peripheral compartment; IIV, interindividual variability; σ, variance of the residual variability, incorporated as an additive error on the logarithmic scale; AUC_0–24_, area under the concentration-time curve from 0 to 24 h; *C*_max_, peak concentration; *T*_max_, time to reach peak concentration; *t*_1/2_, terminal elimination half-life.

b Computed population mean parameter estimates from NONMEM. Parameter estimates are scaled to typical patient at 10.9 kg and 3 years.

c Assessed by sampling importance resampling (SIR).

d Median (range).

### Simulations.

The final population pharmacokinetic models were used to simulate isoniazid, rifampin, pyrazinamide, and ethambutol plasma and CSF exposures at the steady state in children aged 6 months and 1, 2, 5, and 10 years (*n* = 5,000 for each age group). Isoniazid plasma and CSF AUC_0–24_ of 7.03 mg×h/liter (23) and rifampin plasma AUC_0–24_ of 86.4 mg×h/liter (21), corresponding to EC_50_ in adults with TBM, were used as clinical therapeutic targets. More conservative targets (EC_99_) of plasma and CSF AUC_0–24_ of 98.6 mg×h/liter for isoniazid and plasma AUC_0–24_ of 92.0 mg×h/liter for rifampin were also applied. Plasma peak concentrations of 35 mg/liter for pyrazinamide and 2 mg/liter for ethambutol, corresponding to target treatment levels in adults with pulmonary TB, were used as clinical therapeutic targets in this study (17). The probability of target attainment (PTA, i.e., the percentage of virtual children having plasma or CSF exposures above the target exposure) for each drug was calculated in each age group. A PTA >90% was considered to be adequate for tuberculosis treatment.

**FIG 3:**
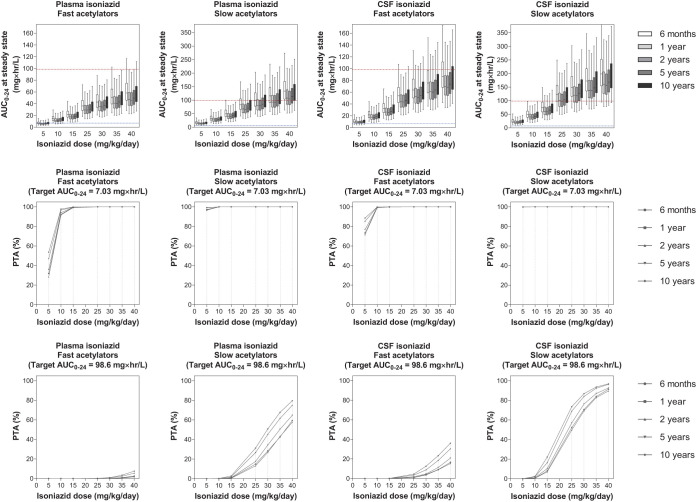
Exposure and probability of target attainment in different isoniazid dosing scenarios. The panels in the top row show box-and-whisker plots (the lower and upper limits of individual boxes denote the 25th and 75th percentiles, and the whiskers represent percentiles 2.5 and 97.5) of simulated steady-state plasma and CSF exposure from 0 to 24 h (AUC_0–24_) of isoniazid. The broken lines represent the plasma/CSF targets of isoniazid; i.e., the lower blue line represents the EC_50_ (7.03 mg × h/liter) and the upper red line represents the EC_99_ (98.6 mg × h/liter) associated with survival in adults with TBM (24). The panels in the two lower rows show the probability of target attainment (PTA) for isoniazid at steady state in plasma and CSF. The shaded bands represent the currently recommended WHO dose of isoniazid.

Our simulations predicted that isoniazid at 10 mg/kg/day, currently recommended by WHO, was adequate, and use of that dose resulted in steady-state plasma and CSF exposure above the EC_50_ target value with PTA >90% for both fast and slow acetylators. However, 40 mg/kg/day of isoniazid was needed to achieve the stricter EC_99_ target for the CSF exposure in patients with slow acetylator status, while even higher doses were needed to reach the CSF target in patients with fast acetylator status. Nevertheless, a higher dose of isoniazid than currently recommended (8 to 12 mg/kg/day) may not be advisable due to risk of hepatotoxicity and peripheral neuropathy (27). Simulated exposures at steady state and the corresponding PTA for each isoniazid dose are presented in Fig. 3.

**FIG 4:**
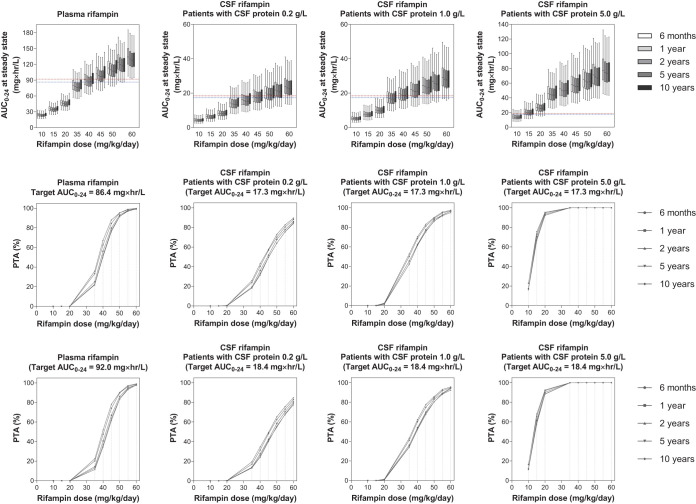
Exposure and probability of target attainment at different rifampin dosing scenarios. The panels in the top row show box-and-whisker plots (the lower and upper limits of individual boxes denote the 25th and 75th percentiles, and the whiskers represent percentiles 2.5 and 97.5) of simulated steady-state plasma and CSF exposure from 0 to 24 h (AUC_0–24_) of rifampin in children with CSF protein content of 0.2, 1.0, and 5.0 g/liter. The broken lines represent the plasma/CSF targets of rifampin; i.e., the lower line represents the EC_50_ (92.0 mg × h/liter in plasma and 18.4 mg × h/liter in CSF), and the upper line represents the EC_99_ (86.4 mg × h/liter in plasma and 17.3 mg × h/liter in CSF) associated with survival in adults with TBM (22). The panels in the two lower rows show the probability of target attainment (PTA) for rifampin at steady state in plasma and CSF. The shaded bands represent the dose of rifampin currently recommended by WHO.

For rifampin, a dose of 50 mg/kg/day was needed to reach the plasma EC_50_ target exposure, and a dose of 55 mg/kg/day was needed to reach the EC_99_ target. An increase in CSF protein concentration indicates a dysfunctional blood-brain barrier, and CSF protein concentration was a significant determinant of rifampin CSF concentration. Therefore, we simulated patients with normal (0.2 g/liter), mild (1.0 g/liter), and severe (5.0 g/liter) blood-brain barrier dysfunction. Simulated exposures at steady state and PTA of each rifampin dose are presented in Fig. 4.

**FIG 5:**
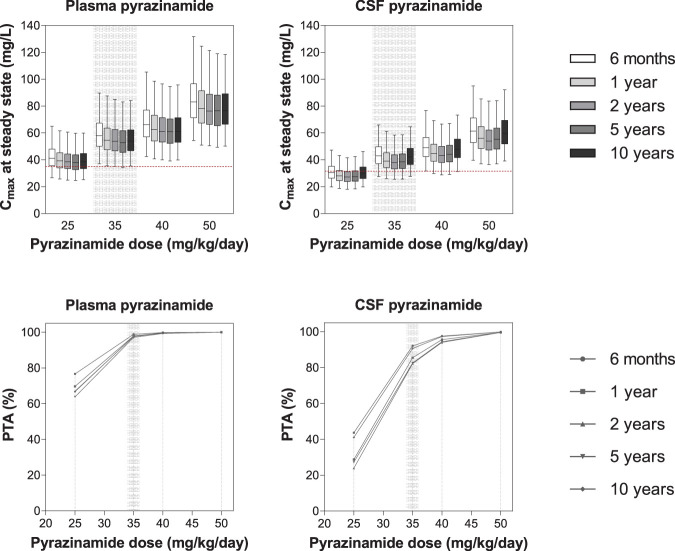
Exposure and probability of target attainment in different pyrazinamide dosing scenarios. The panels in the top row show box-and-whisker plots (the lower and upper limits of individual boxes denote the 25th and 75th percentiles, and the whiskers represent percentiles 2.5 and 97.5) of simulated steady-state plasma and CSF peak concentrations (*C*_max_) of pyrazinamide. The broken red lines represent the plasma/CSF targets of pyrazinamide (31.5 mg/liter) derived from the target for the treatment of pulmonary TB in adults (17). The panels in the bottom row show the probability of target attainment (PTA) for pyrazinamide at steady state in plasma and CSF. The shaded bands represent the dose of pyrazinamide currently recommended by WHO.

The currently recommended dose of pyrazinamide (i.e., 35 mg/kg/day) was adequate to achieve the plasma target peak concentrations with PTA >90%. Nonetheless, the dose should be increased to 40 mg/kg/day to achieve CSF target peak concentrations. Simulated peak concentrations and PTA of each pyrazinamide dose are presented in Fig. 5.

**FIG 6:**
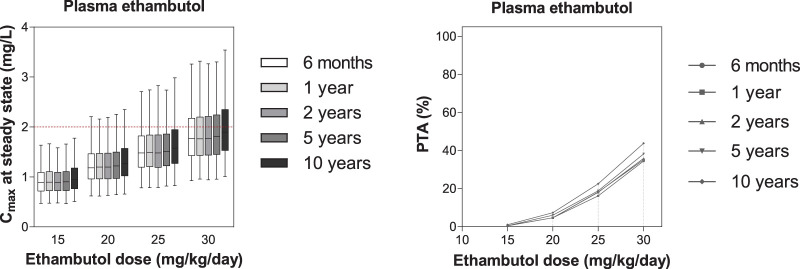
Exposure and probability of target attainment in different ethambutol dosing scenarios. The left panel shows box-and-whisker plots (the lower and upper limits of individual boxes denote the 25th and 75th percentiles, and the whiskers represent percentiles 2.5 and 97.5) of simulated steady-state plasma peak concentrations (*C*_max_) of ethambutol. The broken red line represents the plasma target of ethambutol (2.0 mg/liter) derived from the target for the treatment of pulmonary TB in adults (17). The right panel shows the probability of target attainment (PTA) for ethambutol at steady state in plasma. The shaded bands represent the dose of ethambutol currently recommended by WHO.

Steady-state plasma peak concentrations of ethambutol were below the therapeutic plasma target peak concentration in all the age groups, after simulating ethambutol doses of 15, 20, 25 and 30 mg/kg/day. However, increasing the ethambutol dose to above 25 mg/kg/day is not desirable as ethambutol shows dose-dependent ocular toxicity (28). Simulated peak concentrations and PTA of each ethambutol dose are presented in Fig. 6.

### Population pharmacodynamic analysis.

The treatment outcomes are presented in Table 1. Of the 100 Vietnamese children with TBM who participated in the study, a total of 15 children died over the 4 months of follow-up after the onset of treatment. The majority of these children (53.3%, 8/15) died within the first week of enrollment. The data corresponding to exposure to each of the anti-TB drugs in children who recovered, in children with neurological disability (both intermediate and severe), and children who died are presented in Table S1 in the supplemental material. The results showed that the levels of plasma and CSF exposure at the first day of treatment were not related to the treatment outcomes. However, there was a statistically significant difference (*P* < 0.05) between the rifampin plasma exposure at steady state seen in children that recovered and the exposure seen in children with neurological disability (Table S2).

**FIG 7:**
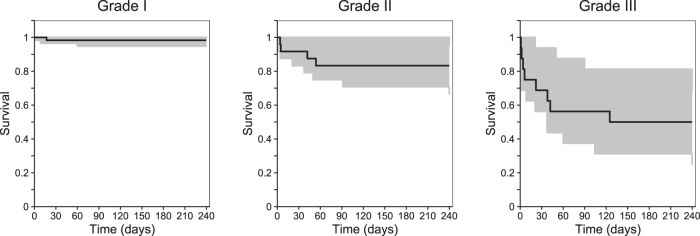
Time-to-event survival analysis, stratified. Visual predictive plots represent the final pharmacodynamic model, stratified by TBM severity (grades I to III). The black lines represent the observed data. Shaded areas represent the 95% prediction intervals for the simulated data (*n* = 1,000). Data were subjected to right-censoring for patients lost to follow-up or at the end of the study (240 days).

The survival data were modeled using a time-to-event (TTE) model. The baseline hazard was best described by a Weibull distribution. Only baseline severity of the TBM infection was identified as a statistically significant covariate on baseline hazard (ΔOFV = −19.0), resulting in a steeper mortality curve for patients with severe disease than for those with mild/moderate disease. The impacts of individual CSF and/or plasma anti-TB drug exposures at the first day of treatment (*C*_max_ or AUC_0–24_) were evaluated in the TTE model, and the results did not indicate a significantly improved model fit. The first-day exposures were used since the majority of deaths were observed during the first week of enrollment. The final pharmacodynamic parameter estimates are summarized in Table S3. Simulation-based diagnoses (pcVPCs) demonstrated a good description of observed survival data and adequate predictive performance of the final model (Fig. 7).

## DISCUSSION

The population PK properties of the four first-line anti-TB drugs were described in Vietnamese children with TBM, especially focusing on the impact of clinical and biochemical determinants and on the optimal dosing of these drugs in this population. All observed plasma and CSF concentration-time profiles were described well with the developed population pharmacokinetic models. Simulations were performed to evaluate optimal dosage regimens of the four anti-TB drugs in these children. Reference steady-state-exposure data from previously published studies (17, 21, 23) were used as clinical therapeutic target exposures in these simulations. Although our results did not support a relationship between anti-TB drug exposures and survival, the results showed that rifampin plasma exposure at steady state was associated with the probability of complete recovery/neurological disability among children who survived. Our results suggested that the dose of rifampin has to be increased to attain the prespecified exposure target of rifampin which would reduce the risk of neurological disability in children. The currently recommended dose of ethambutol was not sufficient to achieve target levels. However, the dose cannot be increased due to ocular toxicity. For isoniazid and pyrazinamide, the currently recommended WHO doses were adequate.

Isoniazid is primarily metabolized to acetyl isoniazid (AcINH) by NAT2 (27). Genetic polymorphisms in NAT2, which result in fast, intermediate, and slow acetylators, have been shown to impact isoniazid plasma concentrations (29, 30). In this study, patients were categorized as fast and slow acetylator phenotypes, based on NAT2 genotype (i.e., fast and intermediate acetylators versus slow acetylators). Only two phenotype groups were used due to the bimodal distribution of observed clearance data and the limited number of patients in the fast acetylator genotype group (*n* = 17). Estimated clearance values of 9.43 liters/h for fast acetylators and 4.11 liters/h for slow acetylators in the current study were similar to the clearance values previously reported in children with pulmonary TB (median age of 2.17 years and median body weight of 12.5 kg), i.e., clearance of 11.6 and 14.6 liters/h for intermediate and fast acetylators and of 4.44 liters/h for slow acetylators (31). These small differences might be due to ethnic differences and/or differences in severity of TB (i.e., TBM versus pulmonary TB). The data representing clearance of isoniazid showed age-dependent maturation, which was consistent with the data previously seen by Zvada et al. (31). The enzyme levels in children aged 12 months in the present study reached 90% of those in adults, while Zvada et al. reported 90% maturation at 24 months. The difference in the estimated enzyme maturation might correspond to ethnic differences, study-specific differences such as sampling design, and/or other unknown population differences. Isoniazid is an ideal drug for treating infections in the central nervous system, due to the small size of the molecule and its lipophilicity. The CSF-to-plasma exposure ratio of isoniazid has been reported to be close to 1 (32, 33). In our study, isoniazid showed a good distribution of drug over the blood-brain barrier, with median CSF-to-plasma ratios of 1.44 for fast acetylators and 1.48 for slow acetylators, resulting in a higher concentration in CSF than in plasma. Isoniazid has been reported to be a substrate of three solute carrier (SLC) transporters, i.e., OCT2, OAT1, and OAT3 (34). These three SLC transporters are expressed in several tissues, including brain tissue and the blood-brain barrier, and typically facilitate uptake of drug substrates (i.e., influx) into the central nervous system (35, 36). Although the *in vivo* function of specific SLC transporters in the blood-brain barrier remains unclear (36), it is possible that isoniazid is actively transported over the blood-brain barrier by these transporters. The activities of drug transporters over the blood-brain barrier are influenced by several factors. For example, age-related maturation of these transporters has been observed (37, 38). The effect of age on blood-brain barrier penetration (PC) was also investigated in the current study, but no significant impact was found. Simulations were performed to evaluate optimal dosing regimens of isoniazid. Simulations of optimal doses demonstrated that 10 mg/kg/day of isoniazid, which is the currently recommended WHO dose, was adequate to maintain steady-state plasma and CSF exposures above the EC_50_ with PTA >90% in both fast and slow acetylators. Similar results were reported in a previous study by Aruldhas et al., who studied pulmonary and lymph node TB in Indian children aged 2 to 16 years (19). However, a study by Zvada et al. showed that the dose of 10 mg/kg/day resulted in inadequate exposure in intermediate and fast acetylators (31). Due to these contradicting results, further studies are necessary to define an appropriate dose of isoniazid in children with different acetylator statuses.

Rifampin is mainly metabolized to 25-deacetyl rifampin, a partly active metabolite, by hepatic esterases (39). Rifampin binds to the nuclear pregnane X receptor (PXR), which activates target genes and the expression of phase I and II drug-metabolizing enzymes and transporters (40). Thus, rifampin induces its own metabolism since PXR also regulates the expression and activity of esterase enzymes in the human liver and intestine (41). This was captured in the model structure by incorporating an enzyme turnover model, resulting in time-dependent clearance of rifampin. The fully induced clearance rate was 80.1% higher than the preinduced clearance rate, and rifampin reached steady-state concentrations within 40 days. The estimated penetration of rifampin from plasma to CSF was poor, likely on account of the high molecular mass (823 Da) and the relatively high level of plasma protein binding (80%) (25, 26, 33). In the present study, only 16.9% (95% CI, 14.5% to 19.6%) of the total drug penetrated the blood-brain barrier, and that value rose 27.8% when the protein content in the CSF increased by 1 g/liter. The levels of CSF penetration of rifampin were reported to be 7.12% in Vietnamese adults with TBM (23) and 5.50% to 8.20% in Indonesian adults with TBM (21, 22). Higher penetration of rifampin into CSF among the children in the present study than seen among adults might be explained by the fact that levels of plasma proteins (both albumin and α_1_-acid glycoprotein) rise with age (42, 43). Consequently, children have a higher fraction of unbound rifampin in plasma, which shifts the equilibrium and results in an overall higher distribution of rifampin from plasma to CSF. The relationship between CSF protein concentration and the distribution of rifampin from plasma to CSF in Vietnamese adults with TBM was described by a linear function, i.e., a 13.9% increase with 1 g/liter CSF protein escalation (23). In Indonesian adults with TBM, the relationship was explained by a linear function with log-transformed CSF protein concentrations, resulting in a 63% increase with each 10-fold increase in CSF protein (22). The relationship between CSF protein levels and plasma-to-CSF distribution of rifampin was expected since CSF protein content is a known marker of CNS inflammation and correlates well with the function of the blood-brain barrier, resulting in higher protein content in the CSF under conditions of severe inflammation due to a leaky blood-brain barrier (33, 44). Consequently, rifampin penetrates the blood-brain barrier better at higher CSF protein concentrations. In healthy persons, spinal fluid normally contains very little protein, resulting in 100% free drug in CSF. Since this study measured only total rifampin levels, there can be no sufficient conclusion about the extent of rifampin protein binding in CSF among meningitis patients.

Estimated clearance and volume of distribution of rifampin in this study were lower than those previously reported for South African children with TB (median age of 2.17 years and median body weight of 12.5 kg) (31). The clearance rate was 3.22 liters/h in this study and was 8.15 liters/h in the South African children with TB, while the volume of distribution was 12.3 liters and 16.2 liters, respectively. This might be explained by ethnic differences or severity of TB or TB-HIV coinfection or by some combination of those factors. Estimated levels of clearance in Vietnamese adult patients with TBM (23) were similar to those estimated in the children studied here and scaled allometrically to 70 kg body weight (i.e., 10.1 liters/h in adults versus 12.9 liters/h in scaled children), supporting the idea of body weight-dependent nonlinear elimination due to increased liver mass per kg and liver blood flow per kg in children compared to adults (45). The estimated age-based maturation of rifampin clearance determined in this study was similar to that described previously by Zvada et al. (31), resulting in 90% fully matured clearance at approximately 36 months in both studies. Previous studies in adults showed that rifampin exhibits concentration-dependent (nonlinear) elimination and dose-dependent absorption properties (7, 46). Neither of the parameters describing these properties was included in the developed model since all children received 10 mg/kg/day of rifampin, resulting in very limited power to estimate these parameters. The addition of nonlinear elimination by fixing the nonlinear elimination parameters (i.e., *K_m_* and *V*_max_) to the previously published values (7) did not improve the model fit, and dose-dependent absorption data cannot be extrapolated directly to children because of very different total doses administered.

Simulations were performed to evaluate optimal dosing regimens of rifampin, and the clinical therapeutic exposure target was found to be associated with survival in a previously published study of adults with TBM (21). Dosing of 15 mg/kg/day of rifampin, the currently recommended WHO dose, was not adequate to achieve PTA >90% in plasma in the children studied here. The dose needed to be increased to 50 mg/kg/day to achieve the plasma target exposure (EC_50_) with PTA >90% and to 55 mg/kg/day to reach the stricter plasma target (EC_99_). Nevertheless, simulations based on data representing Indonesian adults with TBM demonstrated that rifampin 45 mg/kg/day was required to achieve the plasma target exposure of 92 mg×h/liter (i.e., EC_99_) with PTA of 91% (21). Another study investigating Indonesian adults with TBM showed that rifampin 40 mg/kg/day would be required to reach a PTA of around 95% (22). The children studied here required a higher dose of rifampin than adults due to lower plasma rifampin exposures, corresponding to a nonlinear relationship between body weight and drug elimination processes (45). These simulations were also performed with a model incorporating the dose-dependent nonlinear elimination described above, but the results showed a negligible impact on the interpretation of the simulated-dose scenarios (data not shown).

There were several limitations to the final rifampin model. The previously described concentration-dependent (nonlinear) elimination and dose-dependent absorption properties (7, 46) were not included in the final model. In addition to the nonlinear absorption and elimination, autoinduction of rifampin elimination was incorporated by fixing the parameters to previously published data from an adult patient population (6), and enzyme induction in pediatric patients might be different from that in adults. These limitations of the final rifampin model could potentially result in biased simulations when extrapolating beyond the studied doses.

Pyrazinamide is a moderately lipophilic, small molecule with low protein binding (10%) and is therefore expected to exhibit good CSF penetration (33). In our model, the distribution rate was estimated to be 1.02. This suggests that pyrazinamide passes the blood-brain barrier easily by passive diffusion. The clearance and volume of distribution presented in this study were similar to other studies in children and adults with TB (23, 31, 47, 48). The clearance was 1.07 liters/h in this study versus 1.08 liters/h in South African children with TB (median age of 2.17 years and median body weight of 12.5 kg), and the volume of distribution was 7.38 liters in this study versus 9.64 liters in South African children with TB (31). Clearance of pyrazinamide changed with age in the model in accordance with the incorporated maturation factor, and the enzyme levels in children reached 90% of adult levels at the age of 18 months. WAZ was a significant covariate on clearance and central volume of distribution. The clearance decreased 4.76% per unit of WAZ decrease, while the central volume of distribution increased 4.65% per unit of WAZ decrease. The results were inconsistent with those previously reported by Mukherjee et al. (49), who discovered that peak concentrations of and levels of exposure to pyrazinamide were not significantly different between severely malnourished and well-nourished children. The PK/PD target used in the simulations was peak concentration. Simulated pyrazinamide dosing of 35 mg/kg/day, the currently recommended WHO dose, was adequate to achieve PTA >90% in plasma in the children studied here. This was in agreement with a study in South African children (12), estimating PTA to reach 95% after 35 mg/kg of daily pyrazinamide. To increase the efficacy of the treatment of TBM, an increased dose of pyrazinamide (40 mg/kg/day) should be evaluated since this dose was needed to achieve PTA >90% in CSF in the studied population.

For ethambutol, the PK was best described by a two-compartment model with first-order absorption and elimination, and this is consistent with previously published models (23, 48, 50). The clearance seen in this study was higher than that previously reported in adult TB patients (23, 48, 50). The estimated clearance seen in the children studied here (scaled allometrically to 70 kg body weight) was somewhat higher than that seen in Vietnamese adult patients with TBM (23) (i.e., 114 liters/h in scaled children versus 80.3 liters/h in adults), suggesting an additional unknown difference between adults and children that could not be accounted for by body weight and age alone. Age was identified as a covariate on ethambutol clearance, and the enzyme levels reached 90% of adult levels at the age of 30 months. This can be explained by the fact that neonates and infants have a relatively small amount of alcohol dehydrogenase (ADH), the enzyme responsible for ethambutol metabolism. Expression of this enzyme increases with age and does not achieve adult levels until children reach approximately 5 years of age (51). HIV infection was not a significant covariate on any PK parameters in this study, but there were only four HIV-positive children enrolled. This finding contrasted with results seen in the investigations of Zhu et al. (11) and Jönsson et al. (50), who reported that HIV infection was associated with lower ethambutol concentrations. Peak concentration was set as the target for ethambutol. The currently WHO-recommended ethambutol dose, 15 to 25 mg/kg/day, demonstrated suboptimal exposures in the children studied here (PTA <50%). However, an increased ethambutol dose (>25 mg/kg/day) may increase the risk of ocular toxicity and is not advisable (28).

The analysis of the relationship between the treatment outcome and the anti-TB drug exposures showed that steady-state rifampin plasma exposure was significantly higher in the children who recovered fully than in those developing neurological disabilities. This finding supports the suggested increased rifampin dose in TBM patients. However, anti-TB drug exposures were not associated with survival in the time-to-event model. TBM severity was a significant covariate on the estimated baseline hazard of death, resulting in a higher probability of death in patients with TBM that was more severe. The precision of these parameter estimates (see Table S3 in the supplemental material) were relatively high (i.e., >50%) in the patients with baseline severity grades I and II due to the small number of deaths in these two groups (1 death with grade I and 4 deaths with grade II). The absence of a relationship between the anti-TB drug exposures and survival might be explained by the fact that this population PD analysis lacked adequate power (only 15 deaths in total). All children who participated received the same mg/kg dose of the four anti-TB drugs; subsequently, there was limited variability in PK. The survival analyses reported previously by Savic et al. and Svensson et al. showed that rifampin plasma exposure, rather than CSF exposure or plasma peak concentration, was a predictor of survival in patients with TBM. In the plasma exposure models, Savic et al. presented a steady-state rifampin plasma EC_50_ of 86.4 mg×h/liter (21), and Svensson et al. estimated an EC_50_ of 171 mg×h/liter (22). In contrast, the results from the present study showed no significant difference in rifampin plasma exposure at the first day of treatment between dead and surviving patients (median [range] of 34.8 [26.3 to 52.4] versus 37.7 [26.1 to 56.6] mg×h/liter). The rifampin plasma exposure in this study was much lower than the estimated EC_50_ values reported by Savic et al. (21) and Svensson et al. (22), which might explain why an effect was not found here. A time-to-event model recently published by Ding et al. (23) demonstrated that high isoniazid CSF peak concentration and exposure were associated with reduced risk of death in Vietnamese adult patients with TBM; thus, the authors suggested that higher doses of isoniazid should be investigated. Again, such a relationship could not be identified in the patients studied here.

This study had many limitations. First, the PK/PD targets used in the simulations were derived from adults with pulmonary or extrapulmonary TB and might not be directly transferable to children with TBM. The targets for isoniazid and rifampin were based on plasma exposures associated with favorable treatment outcomes in adults with TBM. Peak plasma concentrations of pyrazinamide and ethambutol associated with favorable treatment outcomes in adults with pulmonary TB were used as the targets as no targets are available in TBM patients in the current literature. Second, a quantitative measurement of the level of Mycobacterium tuberculosis in CSF would be a better way to evaluate the efficacy of anti-TB drugs in TBM patients. However, this was not available in the present study and it might be difficult to measure in future studies due to the high level of negative reading results in CSF samples and to the ethical and practical issues associated with collecting regular CSF samples. Third, the children in this study received the same dosage (mg/kg) of the four anti-TB drugs, resulting in limited power of the population PD analysis to detect a possible relationship between the anti-TB drug exposures and survival. Fourth, children were enrolled based on clinical symptoms associated with TBM and we cannot exclude the possibility that a child with suspected TBM was in fact enrolled due to another disease with similar clinical symptoms.

In conclusion, four population PK models were developed to describe the PK properties of the four first-line anti-TB drugs in 100 Vietnamese children with TBM. Modeling and simulations demonstrated that isoniazid dosing at 10 mg/kg/day and pyrazinamide dosing at 35 mg/kg/day were required to achieve the suggested target exposures in at least 90% of children. The currently recommended doses of ethambutol (15 to 25 mg/kg daily) showed suboptimal plasma peak concentrations, but the dose cannot be increased because of ocular toxicity. Rifampin dosing of 50 mg/kg/day was needed to achieve the suggested plasma target exposures in at least 90% of children. Steady-state rifampin plasma exposure was significantly associated with neurological outcome in surviving children; i.e., the chance of complete recovery was increased with increasing rifampin exposure. Higher doses of rifampin should be considered and need to be studied further to establish safety and efficacy in children with TBM.

## MATERIALS AND METHODS

### Patients.

One hundred children admitted with TBM were enrolled into a prospective descriptive study at the Pham Ngoc Thach Hospital for Tuberculosis and Lung Diseases (PNT) in Ho Chi Minh City, Vietnam. Full details of the study design are available in a previously published article (52). Briefly, this study was conducted from October 2009 to March 2011. To be eligible, children had to be aged less than 15 years, have signs and symptoms consistent with TBM (i.e., one or more of clinical presentations of fever, headache, neck stiffness, vomiting, confusion, coma, convulsions, cranial nerve palsies, hemiplegia, or paraplegia), and be suspected by the attending physician of having TBM. At the time of enrollment, all participants were tested for HIV and the severity of suspected TBM was classified using either Blantyre coma score (BCS; children less than 5 years of age) or a modified United Kingdom Medical Research Council criterion based on Glasgow coma score (GCS; children 5 years or older). Blood was drawn from all participants for standard laboratory measurements. In addition, lumbar puncture was carried out to determine white cell count (WCC) and lymphocyte, protein, lactate, and glucose concentrations in CSF.

### Ethics approval and consent to participate.

The study received ethical approval from the ethical review board of Pham Ngoc Thach Hospital, Viet Nam; the Health Services of Ho Chi Minh City; and the Oxford University Tropical Ethics Committee, United Kingdom. Written informed consent was obtained from the parents or guardians of all participants and assent from all children with capacity.

### Treatment regimen.

Children were treated with a once-daily 8-month regimen according to the Vietnamese national treatment guidelines, which are based on the WHO-recommended pediatric treatment regimen from 2006. All participants received isoniazid (5 mg/kg of body weight), rifampin (10 mg/kg), pyrazinamide (25 mg/kg), ethambutol (15 mg/kg), and streptomycin (15 mg/kg) for 2 months. Thereafter, they received isoniazid, rifampin, pyrazinamide, and ethambutol for 1 month (no streptomycin) and isoniazid, rifampin, and ethambutol for the last 5 months (no streptomycin or pyrazinamide). All children received dexamethasone as an adjuvant therapy (52, 53).

### Pharmacokinetic sampling.

For each child, a total of six plasma samples were collected on days 1, 14, 30, and 90. Two plasma samples were randomly drawn at 2 of 10 possible time points (i.e., 1, 2, 3, 4, 5, 6, 8, 12, 18, or 24 h after dose) on each of days 1 and 14, and an additional plasma sample was randomly drawn at 3, 4, or 5 h postdose on each of days 30 and 90. In addition to the plasma samples, two CSF samples were collected within 15 min of plasma collections on days 30 and 90. Plasma and CSF samples were processed and stored at −80°C for drug analysis.

### Drug analysis.

Plasma and CSF concentrations of the anti-TB drugs were quantified using high-performance liquid chromatography-tandem mass spectrometry (LC-MS/MS). Briefly, sample preparation consisted of protein precipitation followed by phospholipid removal using 96-well Phenomenex PHREE plates (Macclesfield, United Kingdom). The four drugs and the corresponding stable isotope-labeled internal standards were analyzed on a ZIC-cHILIC column (Merck Sequant, Umea, Sweden) (5-μm pore size, 50 by 2.1 mm, 3 mm, 100 Å) protected by a ZIC-cHILIC guard column (Merck Sequant, Umea, Sweden) (16 by 1.0 mm, 5 mm). An API 5000 triple-quadrupole mass spectrometer (Applied Biosystems/MDS Sciex) was used with a TurboV ionization source (TIS) interface operated in the positive-ion mode for the multiple-reaction-monitoring LC-MS/MS analysis. The lower limit of quantification (LOQ) was set to 12.0, 8.0, 800, and 8.0 ng/ml for isoniazid, rifampin, pyrazinamide, and ethambutol, respectively, in plasma sample measurements. LOQ was set to 36.0, 32.0, and 200 ng/ml for isoniazid, rifampin, and pyrazinamide, respectively, in CSF sample measurements. Unfortunately, simultaneous quantification of ethambutol was not possible in CSF samples due to separation issues, and the amount of CSF was insufficient for separate quantification of ethambutol in the remaining sample. Three quality control samples were analyzed at high, medium, and low concentrations of all anti-TB drugs within each batch of clinical samples to ensure accuracy and precision during routine clinical sample analysis. Relative standard deviations (RSD) of all quality control samples were less than 15% during clinical trial sample analysis (i.e., within the limit stipulated by the FDA for bioanalytical drug measurements).

### NAT2 genotyping.

The NAT2 gene is polymorphic, resulting in rapid, intermediate, or slow acetylator phenotypes. In the current study, NAT2 genotype was determined for each patient by sequencing the second exon of the NAT2 gene, which contains the functional polymorphisms (54, 55). Genomic DNA was extracted from blood using a Nucleon genomic DNA extraction kit (GE Healthcare, Amersham, United Kingdom). Two primers, NAT2F (5′-TGGGCTTAGAGGCTATTT) and NAT2R (5′-GAGTTGGGTGATACATACAC), were designed using Primer Express version 2.0 software (Applied Biosystems Inc., Foster City, CA, USA) to amplify a 768-bp sequence of the relevant polymorphisms (G191A, C282T, T341C, C481T, G590A, A803G, and G857A) on the NAT2 gene. The phenotypes were predicted from the genotype, using a previously defined correlation between genotype and urinary caffeine metabolite ratio in a cohort of healthy Vietnamese volunteers (53, 56, 57).

### Population pharmacokinetic analysis.

Pharmacokinetic models for the studied drugs were developed using nonlinear mixed-effects modeling (NONMEM version 7; Icon Development Solution, Ellicott City, MD). The first-order conditional estimation method with eta-epsilon interaction (FOCE-I) was used during the model-building process. Postprocessing of the NONMEM output, including graphical diagnostics and simulations, was performed using R version 3.4.4 (The R Foundation for Statistical Computing), Xpose version 4.5.3 (58), and Perl-speaks-NONMEM (PsN) version 4.6.0 (59).

The four anti-TB drug concentrations were converted into natural logarithms, and each drug was modeled separately. Several distribution models, i.e., one-, two-, and three-compartment disposition models, were evaluated during model development. Different absorption models, including first-order absorption with and without lag time and transit compartment models with a fixed number (1 to 10) of transit compartments (60), were also evaluated. Bioavailability was evaluated by fixing it to unity for the population and allowing estimation of interindividual variability (IIV) and interoccasional variability (IOV) in the same parameter. A CSF compartment was incorporated into the developed structural model as illustrated in Fig. 2. The CSF volume was fixed according to the individual age of the patient, based on the published digitized relationship between CSF volume and age (61). This relationship is represented by equation 1 as follows:

(1) $$\text{CSF volume}\left(\text{ml}\right)\text{in children}=150\left[\frac{38.78+\left[\left({\text{age}}^{1.071}\right)\left(102.6-38.78\right)\right]}{{\text{age}}^{1.071}+1.297^{1.071}}\right]$$

where 150 is the adult CSF volume in milliliters (62) and age is expressed in years.

IIV was implemented exponentially in all parameters. IOV was evaluated on relative bioavailability and mean transit time to account for the random within-patient variability between the four different sampling occasions (days 1, 14, 30, and 90). IIV and/or IOV values estimated to be below 10% were fixed to zero. Additive and/or proportional models for the unexplained residual variability were investigated. Drug measurements below the LOQ was coded as missing data (M1 method) when the total amount of drug measurements below the LOQ was less than 5%. If the amount of data below the LOQ was higher than 5%, simulation-based diagnostics (i.e., categorical VPC of the fraction of LOQ data) was used to evaluate potential model misspecifications due to data censoring. If the VPC showed model misspecifications, LOQ data were evaluated with the M3 method for comparison.

An enzyme turnover model was evaluated in the rifampin model because of its autoinduction properties (6). In this model, rifampin concentrations influenced the amount of metabolizing enzyme in the enzyme pool, which subsequently affected the drug clearance (Fig. 2). The change in amount of enzyme over time is expressed by equation 2:

(2) $$\frac{dA_{\text{ENZ}}}{\mathrm{dt}}=k_{{\text{ENZ}}_{\text{in}}}.\left(1+\frac{E_{\text{max}}.C_{p}}{{\text{EC}}_{50}+C_{p}}\right)-\left(k_{{\text{ENZ}}_{\text{out}}}.A_{\text{ENZ}}\right)$$

where *A*_ENZ_ is the relative amount of enzyme in the enzyme pool. $k_{{\text{ENZ}}_{\text{in}}}$ is the zero-order enzyme production rate, and $k_{{\text{ENZ}}_{\text{out}}}$ is the first-order rate constant for the enzyme degradation in the enzyme pool. In the preinduced state, $A_{\text{ENZ}}$ is normalized to 1, and $k_{{\text{ENZ}}_{\text{in}}}$ is set to be equal to $k_{{\text{ENZ}}_{\text{out}}}$. $E_{\text{max}}$ is the maximum enzyme induction, and ${\text{EC}}_{50}$ is the drug concentration that results in half of $E_{\text{max}}$. The relative amount of enzyme ($A_{\text{ENZ}}$) alters the drug clearance according to equation 3:

(3) $$\text{CL}={\text{CL}}_{\text{pre}}.A_{\text{ENZ}}$$

where ${\text{CL and CL}}_{\text{pre}}$ represent induced and preinduced state drug clearance, respectively.

Body weight was added using a fixed allometric function on all plasma and CSF clearance parameters (exponent fixed to 0.75) and plasma volume of distribution (exponent fixed to 1) (63). CSF volume of distribution was fixed according to age regardless of body weight (61). The median body weight of 10.9 kg was used as the reference. Age as a determinant of clearance was evaluated using enzyme maturation effect (MF) as illustrated by equation 4 (63):

(4) $$\text{MF}=\frac{{\text{PMA}}^{\text{HILL}}}{{\text{PMA}}^{\text{HILL}}+{\text{MAT}}_{50}^{\text{HILL}}}$$

where PMA is postmenstrual age, calculated by adding gestation age (assuming full-term gestation at 9.33 months) to the recorded age of the child, and MAT_50_ is the PMA at which the clearance is 50% of the mature clearance. HILL is the Hill coefficient. A reduction in the OFV of >3.84 units was accepted as a statistically significant improvement in model fit at a *P* value of 0.05 for the nested models, with one different degree of freedom for structural model evaluations.

Potentially important clinical covariates, e.g., weight, age, sex, disease severity, renal and liver function tests, nutritional status (weight-for-age z-score [WAZ] and height-for-age z-score [HAZ]), predicted NAT2 phenotypes (only for isoniazid), and central nervous system (CNS) inflammation markers (CSF protein, CSF lactate, CSF glucose, and CSF/blood glucose ratio), were tested using a stepwise covariate model (SCM) building approach. Only statistically significant and biologically plausible covariates were included in the final model if they fulfilled the covariate selection criteria, which were less stringent during the forward addition (*P* value < 0.05, ΔOFV > 3.84) than during the backward elimination (*P* value < 0.01, ΔOFV > 6.63).

Goodness-of-fit (GOF) plots were used to evaluate the model fit. Shrinkage was computed to evaluate the reliability of individual predictions. The predictive performance of the models was evaluated using prediction-corrected visual predictive checks (pcVPCs; *n* = 1,000) (64). Sampling importance resampling (SIR) was used to evaluate parameter uncertainty and model robustness (65).

### Simulations.

Monte Carlo simulations were performed using a total of 5,000 virtual children, aged 6 months or 1, 2, 5, or 10 years, to predict steady-state exposures in plasma and CSF of the four evaluated anti-TB drugs administered at different doses. The investigated dose regimens were (i) the previously recommended dose (WHO 2006), (ii) the currently recommended dose (WHO 2014), (iii) the current maximum recommended dose, and (iv) the proposed increased dose (see Table S4 in the supplemental material). Body weights of simulated subjects were based on the 10th, 25th, 50th, 75th, and 90th percentiles of the observed body weights of the children in the present study at ages 6 months (±2 months, *n* = 30), 1 year (±2 months, *n* = 52), 2 years (±2 months, *n* = 32), 5 year (±1 year, *n* = 38) and 10 years (±1 year, *n* = 16). More details regarding body weight values used in the simulations are shown in Table S5. Thereafter, PTA were calculated in the 5,000 simulated children. The exposure targets for isoniazid and rifampin, associated with favorable treatment outcome, were derived from adults with TBM (21, 23). Isoniazid plasma AUC_0–24_ of 7.03 mg×h/liter (23) and rifampin plasma AUC_0–24_ of 86.4 mg×h/liter (21), corresponding to EC_50_, were used as therapeutic targets. The EC_99_ values from those studies were also used as a stricter therapeutic target for cases in which an aggressive treatment might be acceptable (i.e., higher doses would not lead to serious adverse effects). The EC_99_ values were calculated to be 98.6 mg×h/liter for isoniazid (Hill slope of 1.74) and 92.0 mg×h/liter for rifampin (Hill slope of 118). No pyrazinamide and ethambutol AUC targets associated with treatment outcomes were available in TBM patients. Therefore, plasma peak concentrations associated with treatment outcome in adults with pulmonary TB were used as therapeutic targets. The target plasma peak concentrations were 35 mg/liter for pyrazinamide and 2.0 mg/liter for ethambutol (17). The level of CSF exposure was assumed to be equal to the unbound concentration target in plasma (i.e., unbound fraction × plasma exposure target). The levels of protein binding of isoniazid, rifampin, pyrazinamide, and ethambutol were reported to be 10%, 80%, 10%, and 12%, respectively (24–26), resulting in a CSF AUC_0–24_ target of >17.3 mg×h/liter for rifampin and a CSF peak concentration target of >31.5 mg/liter for pyrazinamide.

### Population pharmacodynamic analysis.

A time-to-event model was used to characterize the time to death. Patients were followed for the entire treatment period of 8 months. Survival with and without neurological disability was assessed using the modified Rankin scale at the end of the 8-month treatment. The scores ranged from 0 (complete recovery) to 6 (death) (66). The relationship between the 4 anti-TB drug exposures and treatment outcome (full recovery, neurological disability, or death) was evaluated using the Mann-Whitney test.

The time-to-death model was developed using several hazard models and a cumulative hazard function (i.e., exponential, Weibull, and Gompertz functions) (67). All biologically plausible covariates (e.g., age, body weight, WAZ, HAZ, baseline TBM severity, HIV status, C-reactive protein [CRP], CSF protein, CSF lactate, CSF glucose, and CSF/blood glucose ratio) were evaluated with a stepwise covariate approach (as defined for the population PK models). Values representing the potential influence of drug exposures (*C*_max_ or AUC_0–24_, during the first day of treatment) were derived using the final population PK model and evaluated using an exponential model or sigmoidal maximum enzyme induction (*E*_max_) model with or without estimated Hill coefficient.

### Data availability.

All relevant NONMEM code for the pharmacokinetic and pharmacodynamic models is available from the authors upon request and is also freely available at the DDMoRe Model Repository (http://repository.ddmore.eu/models). Due to ethical and security considerations, the underlying patient data that support the findings in this study can be accessed only through the Data Access Committee at Mahidol Oxford Tropical Medicine Research Unit (MORU). The data sharing policy can be found at http://www.tropmedres.ac/data-sharing.

## Supplementary Material

Supplemental file 1
