# Supplementary material for: Population Pharmacokinetic Properties of Antituberculosis Drugs in Vietnamese Children with Tuberculous Meningitis
Source: Antimicrob Agents Chemother. 2020 Dec 16;65(1):e00487-20. doi: 10.1128/AAC.00487-20 (PMC7927832; doi:10.1128/AAC.00487-20)
Supplement: Supplemental file 1 [file AAC.00487-20-s0001.pdf]

Supplementary material

Table S1. The exposures of each anti-TB drug at the first day of treatment stratified for outcome; full recovery, neurological disability (both intermediate and severe) and death.

Anti-TB drugs	Fully recovered children (n = 54)	Children with neurological disability (n = 27)	Death (n = 14)	P-value
**Isoniazid**				
Plasma AUC_{0-24} (mg×hr/L)	7.21 (3.55-19.3)	7.32 (3.24-29.1)	6.83 (5.24-24.2)	0.948
Plasma C_{max} (mg/L)	2.03 (1.55-4.89)	2.29 (1.46-5.04)	2.18 (1.69-2.84)	0.559
CSF AUC_{0-24} (mg×hr/L)	10.7 (5.27-28.7)	10.9 (4.80-43.2)	10.1 (7.78-35.9)	0.948
CSF C_{max} (mg/L)	3.02 (2.30-7.26)	3.39 (2.17-7.48)	3.24 (2.51-4.21)	0.559
**Rifampicin**				
Plasma AUC_{0-24} (mg×hr/L)	37.6 (26.3-56.6)	38.5 (26.1-55.7)	34.8 (26.3-52.4)	0.455
Plasma C_{max} (mg/L)	5.96 (3.95-9.46)	6.30 (3.46-8.49)	6.20 (3.09-8.70)	0.972
CSF AUC_{0-24} (mg×hr/L)	4.45 (2.44-13.2)	5.05 (2.73-7.30)	5.13 (2.36-10.4)	0.745
CSF C_{max} (mg/L)	0.245 (0.131-0.760)	0.276 (0.155-0.391)	0.280 (0.131-0.562)	0.822
**Pyrazinamide**				
Plasma AUC_{0-24} (mg×hr/L)	298 (172-586)	286 (237-553)	313 (178-457)	0.762
Plasma C_{max} (mg/L)	35.8 (23.9-55.5)	38.2 (26.6-60.0)	38.2 (24.6-48.8)	0.366
CSF AUC_{0-24} (mg×hr/L)	274 (157-532)	262 (216-532)	284 (163-414)	0.767
CSF C_{max} (mg/L)	27.0 (17.2-46.3)	27.9 (19.6-46.1)	26.6 (17.1-36.0)	0.774
**Ethambutol**				
Plasma AUC_{0-24} (mg×hr/L)	7.31 (4.92-27.4)	7.26 (4.38-35.4)	7.26 (3.29-8.70)	0.832
Plasma C_{max} (mg/L)	1.12 (0.593-2.26)	1.12 (0.541-2.55)	1.08 (0.431-1.31)	0.449

Data are reported as median (range). The Kruskal-Wallis test was used to evaluate differences among the three groups of children. Abbreviations: AUC_{0-24}, area under the concentration-time curve from 0 to 24 hours; C_{max}, peak concentration. The results showed that plasma and CSF exposures at the first day of treatment was not related to the treatment outcomes.

Table S2. The exposures of each anti-TB drug at steady-state stratified for outcome; full recovery and neurological disability (both intermediate and severe).

Anti-TB drugs	Fully recovered children (n = 54)	Children with neurological disability (n = 27)	P-value
**Isoniazid**			
Plasma $AUC_{0\text{-}24}$ (mg×hr/L)	6.70 (3.09-17.9)	7.79 (2.64-21.6)	0.237
Plasma C_{max} (mg/L)	2.12 (1.59-4.89)	2.36 (1.47-5.13)	0.129
CSF $AUC_{0\text{-}24}$ (mg×hr/L)	9.94 (4.59-26.6)	11.0 (3.91-32.1)	0.482
CSF C_{max} (mg/L)	3.15 (2.36-7.26)	3.51 (2.18-7.61)	0.130
**Rifampicin**			
Plasma $AUC_{0\text{-}24}$ (mg×hr/L)	22.6 (17.3-33.0)	20.2 (14.2-36.5)	0.0137**
Plasma C_{max} (mg/L)	5.13 (2.89-8.37)	4.89 (2.46-6.98)	0.309
CSF $AUC_{0\text{-}24}$ (mg×hr/L)	4.06 (2.59-9.34)	4.40 (2.91-8.29)	0.518
CSF C_{max} (mg/L)	0.244 (0.161-0.518)	0.281 (0.176-0.559)	0.180
**Pyrazinamide**			
Plasma $AUC_{0\text{-}24}$ (mg×hr/L)	296 (137-665)	278 (120-527)	0.469
Plasma C_{max} (mg/L)	40.9 (30.5-73.1)	44.0 (29.8-76.4)	0.523
CSF $AUC_{0\text{-}24}$ (mg×hr/L)	272 (126-609)	256 (111-485)	0.468
CSF C_{max} (mg/L)	31.0 (19.4-72.2)	32.0 (22.4-60.3)	0.954
**Ethambutol**			
Plasma $AUC_{0\text{-}24}$ (mg×hr/L)	7.68 (4.96-17.8)	8.22 (5.03-12.1)	0.617
Plasma C_{max} (mg/L)	1.25 (0.700-2.54)	1.29 (0.656-2.30)	0.594

** Statistically significant difference at p-value<0.05. Data are reported as median (range). The Mann-Whitney test was used to evaluate differences between children with full recovery and neurological disability. Abbreviations: $AUC_{0\text{-}24}$, area under the concentration-time curve from 0 to 24 hours; C_{max}, peak concentration. Note that there was a statistically significant difference between rifampicin plasma $AUC_{0\text{-}24}$ at steady-state in children with full recovery and children with neurological disability.

Table S3. Final parameter estimates of the time-to-event (death) model.

Parameters	Population estimates[a] (%RSE[b])	95%CI[b]
Baseline (hr^{-1})		
Grade I	$5.74×10^{-9}$ (73.8)	$6.38×10^{-17}$ - $1.34×10^{-8}$
Grade II	$2.35×10^{-6}$ (87.0)	$1.73×10^{-10}$ - $2.87×10^{-6}$
Grade III	$7.94×10^{-5}$ (11.7)	$5.70×10^{-6}$ - $4.84×10^{-4}$
Slope	0.391 (15.3)	0.304 - 0.545

[a] Computed population mean parameter estimates from NONMEM. [b] Assessed by bootstrap. The hazard function was described by the equations below.

$$h(t) = \lambda\alpha(\lambda t)^{\alpha-1},\ \lambda = \left(10^{\theta}\right)(TBM\ Severity)$$

where $h(t)$ is the hazard function; λ is a scale factor; α is a shape factor; t is the survival time; θ is the typical value of the baseline hazard.

Table S4. Doses of anti-tuberculosis drugs used in Monte Carlo simulations

Anti-TB drugs	Doses used in the simulation (mg/kg/day)			
	Previously recommended dose[a] (WHO 2006)	Currently recommended dose (WHO 2014)	Currently recommended maximum dose (WHO 2014)	Proposed new dose
Isoniazid	5	10	15	25-40
Rifampicin	10	15	20	35-60
Pyrazinamide	25	35	40	50
Ethambutol	15	20	25	30

[a] The TB treatment regimen used in this study

Table S5. Body weight used in the simulations

Age (kg) / Percentiles	6 months	1 year	2 years	5 years	10 years
10th	4.50	6.62	8.64	10.7	16.5
25th	6.08	7.20	9.58	13.1	17.4
50th	6.90	8.50	10.5	14.5	20.2
75th	8.68	9.42	11.8	15.6	26.0
90th	9.05	11.0	12.5	17.2	29.5

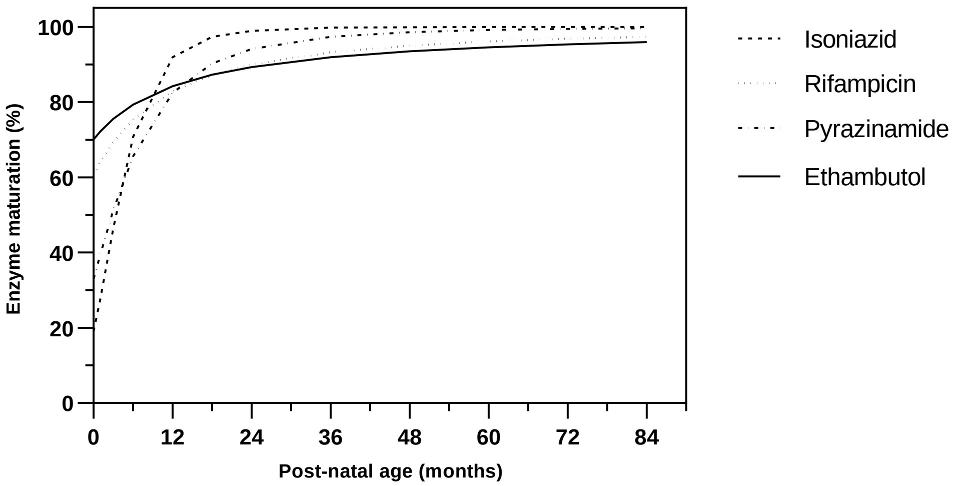

Figure S1. Age-based maturation of clearance of the four anti-TB drugs in a typical patient. The age of children in the study range from 2 months to 15 years.

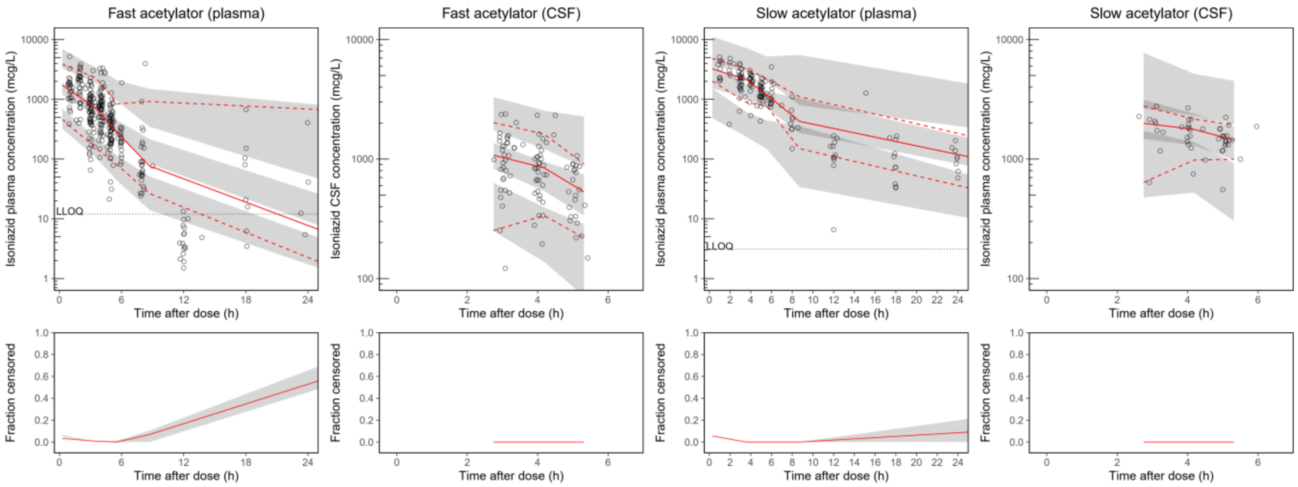

Figure S2. Prediction-corrected visual predictive check of the final isoniazid population pharmacokinetic model stratified on acetylator status (fast vs slow) and sampling matrix (plasma vs CSF). Open circles represent the observed data. The lower, middle and upper lines are the 5th, 50th and 95th percentiles of the observed data. The shaded areas are the 95% confidence intervals of the 5th, 50th and 95th percentiles of the simulated data (n=1,000).

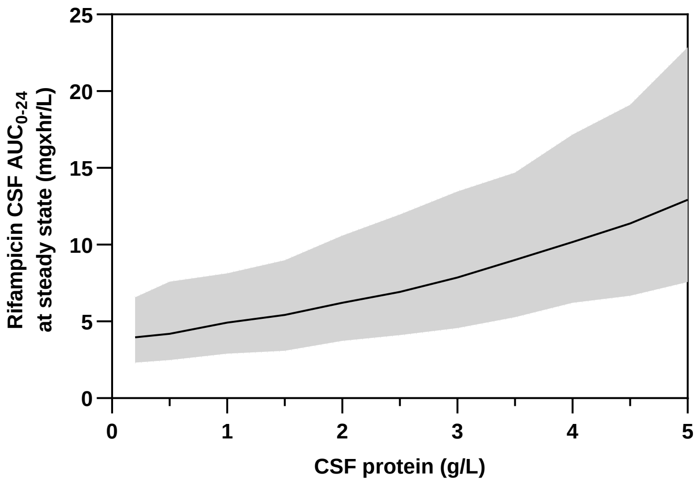

Figure S3. Simulated rifampicin CSF exposure from 0 to 24 hours (AUC_{0-24}) at steady-state versus CSF protein concentrations in children 2 years of age (n=1,000). The black line is the median for the simulated data and shaded area is the 2.5-97.5th percentile range of the simulated data.
